# COVID-19 associated mortality and cardiovascular disease outcomes among US women veterans

**DOI:** 10.1038/s41598-021-88111-z

**Published:** 2021-04-19

**Authors:** Shirling Tsai, Hang Nguyen, Ramin Ebrahimi, Monica R. Barbosa, Bala Ramanan, Daniel F. Heitjan, Jeffrey L. Hastings, J. Gregory Modrall, Haekyung Jeon-Slaughter

**Affiliations:** 1 Veterans Affairs North Texas Health Care System, Dallas, TX USA; 2 Department of Surgery, University of Texas Southwestern Medical Center, Dallas, TX USA; 3 Southern Methodist University, Dallas, TX USA; 4 Veterans Affairs Greater Los Angeles Health Care System, Los Angeles, CA USA; 5 Department of Medicine, University of California at Los Angeles, Los Angeles, CA USA; 6 Department of Internal Medicine, University of Texas Southwestern Medical Center, Dallas, TX USA; 7 Department of Population and Data Sciences, University of Texas Southwestern Medical Center, Dallas, TX USA

**Keywords:** Epidemiology, Risk factors, Infectious diseases

## Abstract

The burden of COVID-19 has been noted to be disproportionately greater in minority women, a population that is nevertheless still understudied in COVID-19 research. We conducted an observational study to examine COVID-19-associated mortality and cardiovascular disease outcomes after testing (henceforth index) among a racially diverse adult women veteran population. We assembled a retrospective cohort from a Veterans Affairs (VA) national COVID-19 shared data repository, collected between February and August 2020. A case was defined as a woman veteran who tested positive for SARS-COV-2, and a control as a woman veteran who tested negative. We used Kaplan–Meier curves and the Cox proportional hazards model to examine the distribution of time to death and the effects of baseline predictors on mortality risk. We used generalized linear models to examine 60-day cardiovascular disease outcomes. Covariates studied included age, body mass index (BMI), and active smoking status at index, and pre-existing conditions of diabetes, chronic kidney disease (CKD), chronic obstructive pulmonary disease (COPD), and a history of treatment with antiplatelet or anti-thrombotic drug at any time in the 2 years prior to the index date. Women veterans who tested positive for SARS-CoV-2 had 4 times higher mortality risk than women veterans who tested negative (Hazard Ratio 3.8, 95% Confidence Interval CI 2.92 to 4.89) but had lower risk of cardiovascular events (Odds Ratio OR 0.78, 95% CI 0.66 to 0.92) and developing new heart disease conditions within 60 days (OR 0.67, 95% CI 0.58 to 0.77). Older age, obesity (BMI > 30), and prior CVD and COPD conditions were positively associated with increased mortality in 60 days. Despite a higher infection rate among minority women veterans, there was no significant race difference in mortality, cardiovascular events, or onset of heart disease. SARS-CoV-2 infection increased short-term mortality risk among women veterans similarly across race groups. However, there was no evidence of increased cardiovascular disease incidence in 60 days. A longer follow-up of women veterans who tested positive is warranted.

## Introduction

The short-term outcomes of Coronavirus disease 2019 (COVID-19), the disease resulting from infection by the novel severe acute respiratory syndrome coronavirus 2 (SARS-CoV-2), on women, especially minority women, is an area in need of heightened focus and investigation. While the socio-economic effects of COVID-19 on women have been publicized in the mainstream media^1,2^, the extent of the health burden, in terms of mortality and cardiovascular morbidity, remains understudied and poorly understood. While earlier studies showed that men were at higher risk of mortality after contracting COVID-19 than women^3–6^, older women and minority women are also at increased risk of mortality^7–10^. In addition to mortality, COVID-19 patients are at elevated risk of cardiovascular complications^11,12^. Furthermore, pre-existing cardiovascular disease and chronic disease, such as diabetes and COPD^13,14^, have been linked to worse prognosis in COVID-19^12,15^.

The United States (US) Veteran Health Administration (VHA) is the largest integrated healthcare system in the US. Although the VHA data is focused on veteran health, results of large studies based on Veterans Affairs (VA) data have been extrapolated to the general population and now constitute the bases of clinical practice guidelines and standards of care^16–18^. The VHA electronic health record (EHR) provides a rich longitudinal database for the analysis of short-term and longer-term outcomes. The US VA COVID-19 shared repository is a part of VA Infrastructure and Computing Infrastructure (VINCI) Corporate Data Warehouse (CDW) and available to VA researchers. The VA COVID-19 shared repository includes patient-level data on COVID testing, diagnoses, drug utilization from COVID-19 National Bio-Surveillance team and a comprehensive data set of patient demographic and clinical characteristics, treatment, laboratory results, pharmacological therapy, and any adverse events such as death and cardiovascular disease events from VHA EHR.

The purpose of this study was to examine the effect of COVID-19 on women veterans, and specifically minorities, because previous studies have demonstrated that COVID-19 has affected veterans of racial and ethnic minorities disproportionately^3^. The women veteran population has a greater number of cardiovascular disease risk factors than the general population^19^, and their aging trajectory of increased cardiovascular disease (CVD) risk starts as young as age 30^20^. In light of these findings, the women veteran population may be at higher risk of COVID-19-associated mortality and cardiovascular complications than the general population.

This study capitalized on a large number of women representing racial and ethnic minorities in the women veteran population. With a study cohort comprised of 33% African Americans and 8% Hispanics, the current study examined the effect of COVID-19 on mortality and cardiovascular risk in women veterans within 60 days of testing positive using data from the US VA COVID-19 shared data repository, collected between February 24 and November 25, 2020. Notably, most previous studies reporting a lower risk among females rely on data collected up to June 2020. The study findings include data from the second and the third waves of the COVID-19 pandemic in the US (between July 2020 and November 2020), and therefore provide more insight than previous studies on the effect of COVID-19 on short-term mortality and cardiovascular disease outcomes among women.

## Results

We analyzed data from 77,364 women veterans who were tested for SARS-CoV-2 infection at VA hospitals and clinics between February 24, 2020 and November 25, 2020. The mean age of the women veterans was 50.69 ± 12.80 years. Approximately 50% (49.44%) of the women veterans were Caucasian; 32% were African American and 9% were Hispanic (Table 1). In terms of co-morbidities, 19% had diabetes, 19% had a previous diagnosis of cardiovascular disease, and 11% had been diagnosed with COPD. Approximately 11% of the women (n = 8308) tested positive (confirmed and presumptively positive) for SARS-CoV-2.

**Table 1:** Baseline characteristics and 60 day outcomes of veteran women stratified by COVID-19 testing result, positive (+) and negative (−).

Baseline characteristics at index		All (n = 77,364)	Positive (+) (n = 8308)	Negative (−) (n = 69,056)	P-value^a^
Age at index	Mean $\pm$ SD	50.69 $\pm$ 12.80	48.62 $\pm$ 12.66	50.94 $\pm$ 12.80	< 0.001
BMI at index	Mean $\pm$ SD	30.72 $\pm$ 6.88	31.67 $\pm$ 6.84	30.61 $\pm 6$.88	< 0.001
**Race**					
Black	n (%)	24,703 (31.93)	3086 (37.14)	21,617 (31.30)	< 0.001
Hispanics	n (%)	6611 (8.55)	882 (10.62)	5729 (8.30)	
White	n (%)	39,507 (51.07)	3688 (44.39)	35,819 (51.87)	
Other	n (%)	6543 (8.46)	652 (7.85)	5891 (8.53)	
**DM**					
Yes	n (%)	14,667 (19.0)	1591 (19.1)	13,076 (18.9)	0.64
**Current smoker**					
Yes	n (%)	12.259 (15.85)	698 (8.40)	11,561 (14.94)	< 0.001
**CVD**					
Yes	n (%)	14,908 (19.27)	1351 (16.26)	13,557 (19.63)	< 0.001
**COPD**					
Yes	n (%)	8501 (10.99)	624 (7.51)	7877 (11.41)	< 0.001
**CKD**					
Yes	n (%)	3915 (5.06)	347 (4.18)	3568 (5.17)	< 0.001
**Anticoagulant**					
Yes	n (%)	10,133 (13.10)	907 (10.91)	9226 (13.36)	< 0.001
**60 day outcomes**					
Death					
Yes	n (%)	321 (0.41)	79 (0.95)	242 (0.35)	< 0.001
CVD					
Yes	n (%)	2232 (2.89)	166 (2.00)	2066 (2.67)	< 0.001
Ischemic stroke					
Yes	n (%)	703 (0.90)	48 (0.58)	655 (0.95)	< 0.001
Hemorrhagic stroke					
Yes	n (%)	44 (0.06)	4 (0.05)	40 (0.06)	0.72
Troponin > 0.4 mg/dL					
Yes	n (%)	87 (0.11)	15 (0.18)	72 (0.10)	0.06
Heart failure					
Yes	n (%)	1606 (2.08)	117 (1.41)	1489 (2.16)	< 0.001
**New diagnosis in 60 days from index**					
New CVD					
Yes	n (%)	2161 (2.79)	151 (1.82)	2010 (2.60)	< 0.001
New CAHD					
Yes	n (%)	790 (1.02)	46 (0.55)	744 (1.081)	< 0.001
New cardiomyopathy					
Yes	n (%)	164 (0.21)	13 (0.16)	151 (0.20)	0.24
New cerebrovascular disease					
Yes	n (%)	183 (0.24)	10 (0.12)	173 (0.25)	0.02
New hypertension					
Yes	n (%)	1169 (1.51)	109 (1.31)	1060 (1.53)	0.12
New other ill-defined heart disease					
Yes	n (%)	296 (0.38)	28 (0.34)	268 (0.35)	0.48

BMI: Body Mass Index; CAHD : coronary artery heart disease; CVD: cardiovascular disease; DM: Diabetes; CKD: Chronic Kidney Disease; COPD: Chronic Obstructive Pulmonary Disease; HR: Hazard Ratios; CI: Confidence Intervals.

^a^T and chi-squared tests were used for continuous and categorical characteristics, respectively.

eFigure 1 in Supplement depicts the proportions of test-positive women veterans by state. While the total number of women veterans who were tested are highest from VA facilities located in California and New York, the proportion of SARS-CoV-2-positive patients was disproportionately higher in southern states including Texas, Louisiana, Alabama, Georgia, and South Carolina, as well as in New Jersey and Nevada. The number of test-positive women veterans at VA reached an initial peak during July 2020, at which time the number of tests in women veterans was the highest, but the number of test-positive women veterans continued to rise in November 2020 to surpass the number of test-positive women veterans in July 2020 (eFigure 2 in Supplement). There were 79 COVID-19 associated deaths among women veterans. Women veterans living in Texas had the highest numbers of COVID-19-associated death within 60 days of a positive test (eFigure 3 in Supplement).

Women veterans who were SARS-CoV-2-positive were significantly younger but had a higher BMI than those who tested negative (Table 1). Significantly higher numbers of women veterans with minority ethnicity tested positive. While similar to SARS-CoV-2-negative women veterans in terms of prevalence of diabetes, the SARS-CoV-2-positive women veterans were less likely to be current smokers, less likely to have been diagnosed with CVD, CKD, or COPD prior to the index date, and less likely to have been prescribed an antiplatelet or antithrombotic medication in the 2 years prior to the index date (Table 1). Overall, women veterans who tested positive for SARS-CoV-2 were healthier than their test-negative counterparts at the index date.

**Figure 1:**
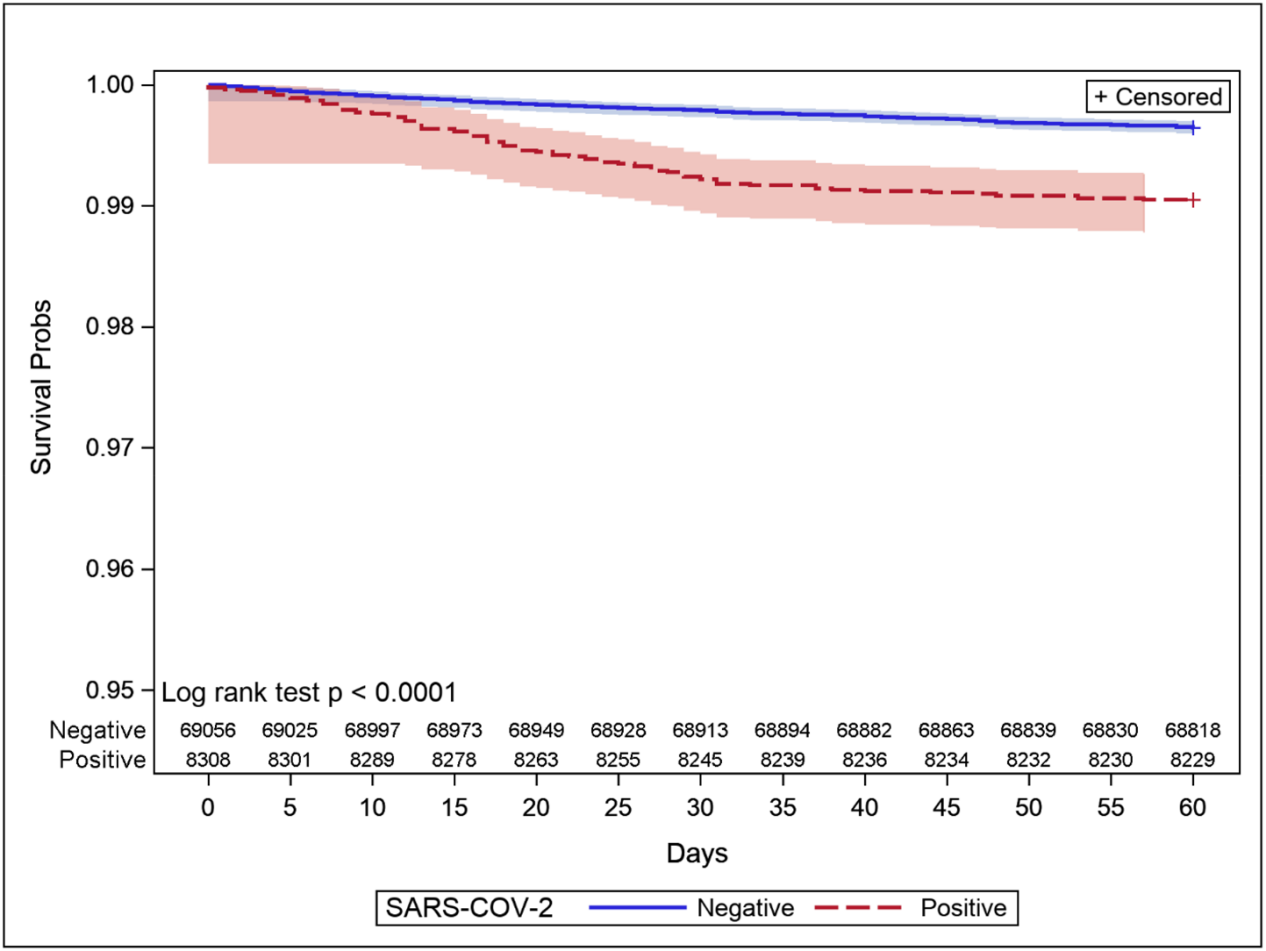
Time to death: Kaplan–Meier curves between SARS-CoV-2-positive and negative groups.

Kaplan–Meier curves showed that SARS-CoV-2-positive women veterans were at higher risk of 60 day mortality risk than SARS-CoV-2-negative women veterans (Fig. 1). SARS-CoV-2-positive women veterans were at nearly 4 times (Hazard Ratio HR 3.8, 95% Confidence Interval CI 2.92 to 4.89) higher risk of death within 60 days than SARS-CoV-2-negative women veterans.

We tested whether age and BMI at index were linearly associated with 60-day mortality risk by fitting generalized additive models^21^ with splines with two degrees of freedom. The test results confirmed that the effect of age on mortality risk was linear (χ^2^ = 1.87; p = 0.17), while the effect of BMI was non-linear with a change in the rate occurring at a BMI value of 30 (χ^2^ = 42.22; p < 0.001; eFigure 4 in Supplement). Thus, the BMI covariates included both the BMI level and an indicator of BMI > 30 additionally to capture this nonlinearity.

Older age (HR 1.07, 95% CI 1.06 to1.09) and BMI in excess of 30 (HR 1.20, 95% CI 1.15 to1.25) were independently associated with increased 60-day mortality risk among all women veterans and also the SARS-CoV-2-positive group (Older age HR 1.10, 95% CI 1.07 to 1.13; BMI > 30 h 1.15, 95% CI 1.05 to 1.25). The pre-existing conditions of CVD (HR 1.50, 95% CI 1.15 to 1.96), COPD (HR 1.38, 95% CI 1.06 to 1.78), CKD (HR 1.55, 95% CI 1.16 to 2.88), and prior antiplatelet or antithrombotic therapy (HR 2.21, 95% CI 1.69 to 2.88) significantly increased the risk of death at 60 days after the index date for all women veterans who underwent testing, while only CVD (HR 2.04, 95% CI 1.18 to 3.52) and COPD (HR 1.81, 95% CI 1.08–3.06) remained significantly associated with increased risk of 60-day mortality among the SARS-CoV-2-positive group. The pre-existing CKD condition was positively associated with 60-day mortality among the SARS-CoV-2-positive group but with attenuated statistical significance (HR 1.65, 95% CI 0.92 to 2.93. p = 0.09). Prior antiplatelet or antithrombotic therapy was no longer associated with mortality risk among the SARS-CoV-2-positive group.

**Figure 2:**
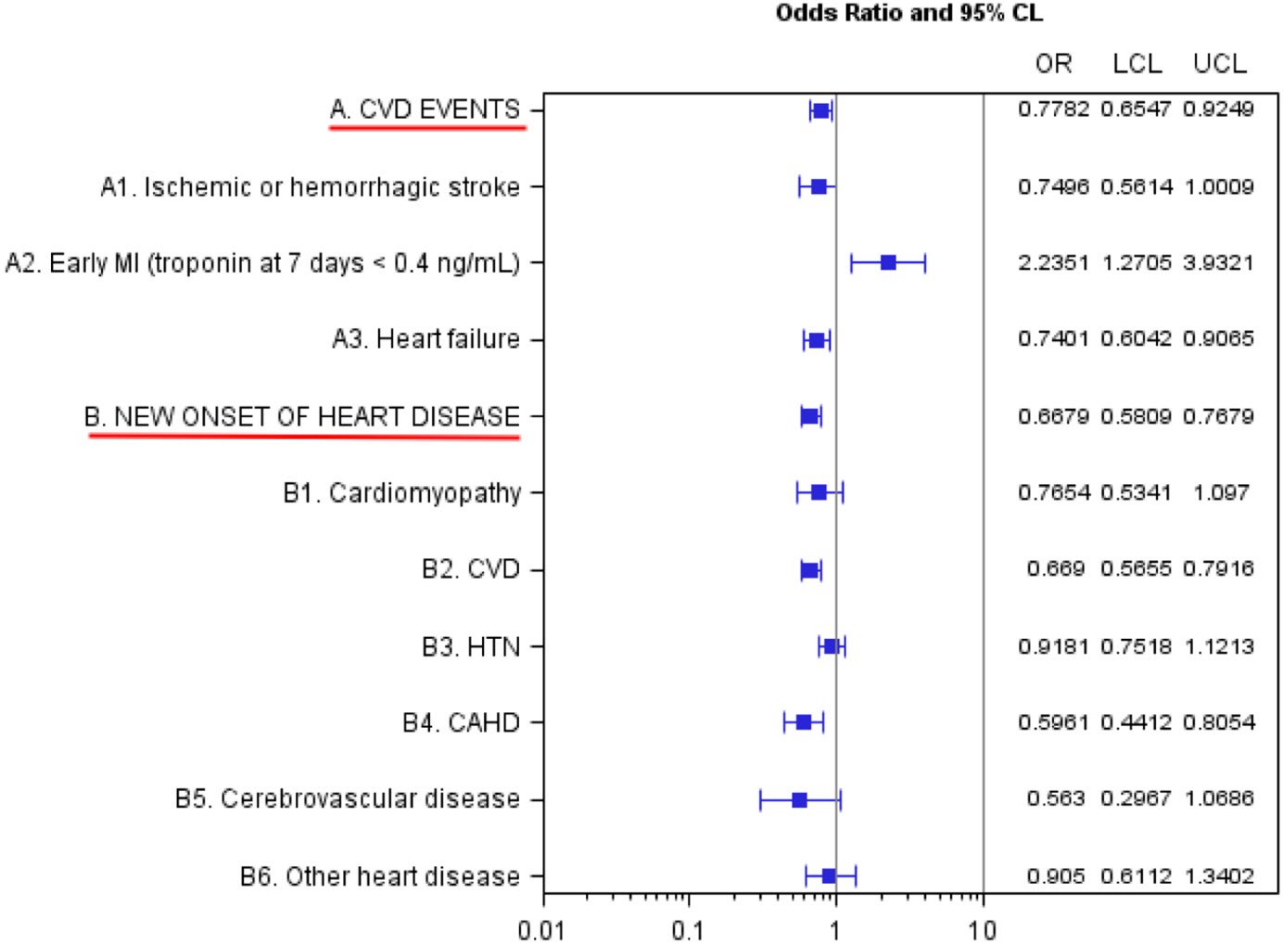
Estimated odds ratios of 60 day cardiovascular outcomes after SARS-CoV-2 exposure. CAHD: Coronary artery Atherosclerosis Disease; CKD: Chronic Kidney disease; CVD: cardiovascular disease; MI: Myocardial Ischemia; HTN: Hypertension; OR = Odds Ratios; CI: Confidence Intervals. a. Adjusted for age at index, race, diabetes, current smoking status, CVD, COPD, CKD, and anticoagulant medication. b. Adjusted for age at index, race, diabetes, current smoking status, COPD, CKD, and anticoagulant medication. c. CVD events included subcategories of events such as Ischemic or hemorrhagic stroke at 60 days, early MI at 7 days, and Heart failure at 60 days. All CVD events are withing 60 days from the index date except early MI which is within 7 days. d. New onset of heart disease included subcategories of heart disease such as Cardiomyopathy, CVD, HTN, CAHD, Cerebrovascular disease, and Other heart disease. e. A 95% CI excluding 1 indicates statistical significance at α: 0.05.

Women veterans who tested positive were at a significantly elevated risk of early myocardial ischemia (OR 2.23, 95% CI 1.27 to 3.94; Fig. 2), as defined by an elevation in troponin at 7 days after index. However, testing positive for SARS-CoV-2 was associated with a reduced risk of experiencing the combined endpoint of any CVD event (including ischemic or hemorrhagic stroke, early MI, and heart failure) within 60 days of the index date (OR 0.78, 95% CI 0.66 to 0.92; Fig. 2). Moreover, there was no significant impact on the risk of new onset heart disease at 60 days (Fig. 2).

There was no significant difference between race groups in the rate of CVD events or new onset of heart disease at 60 days post-test.

**Figure 3:**
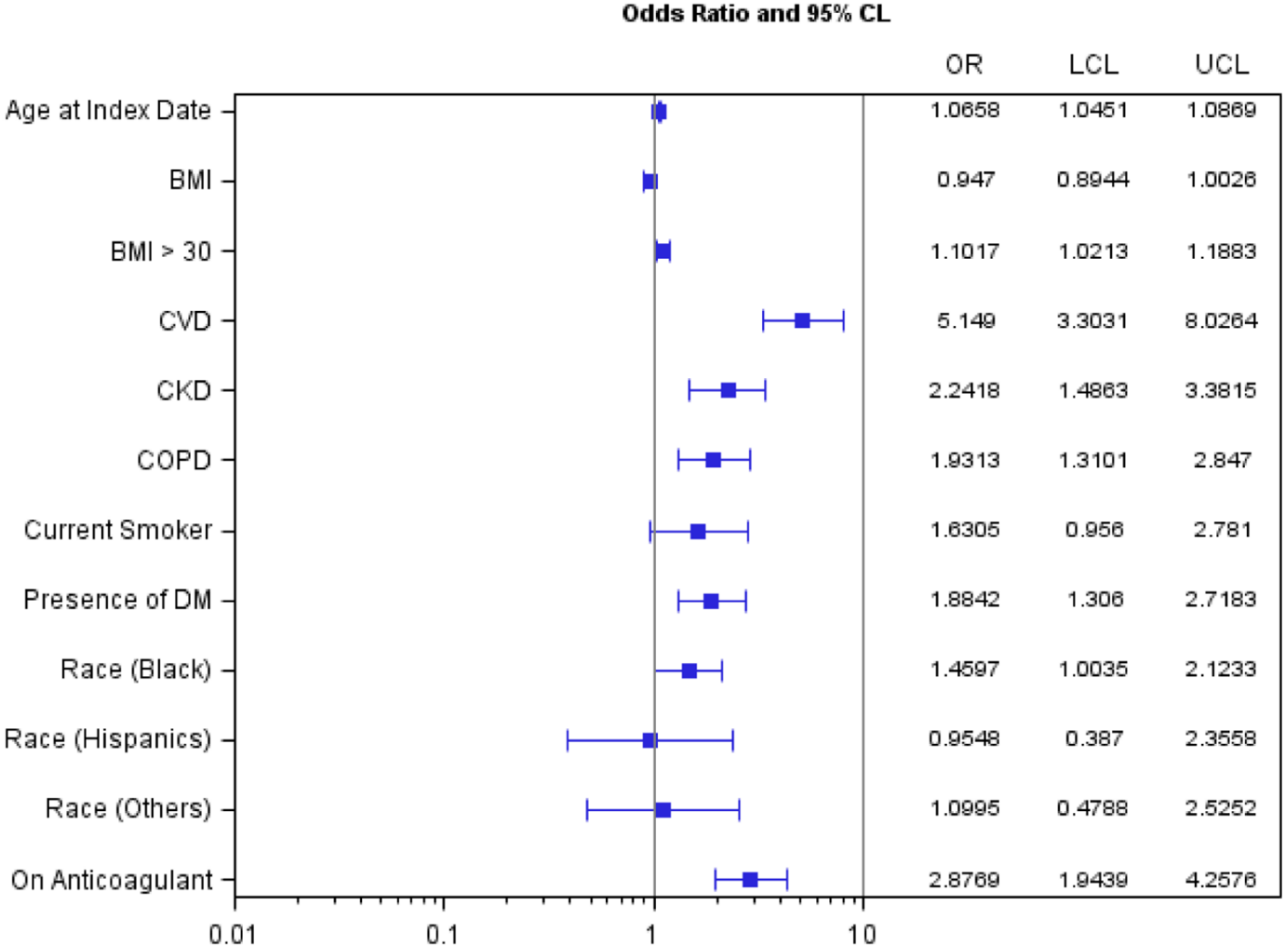
Odds ratios (OR) and 95% confidence interval estimates of risk factors for 60-day cardiovascular events among SARS-CoV-2-positive women veterans. BMI: Body Mass Index; CKD: Chronic Kidney Disease; COPD: Chronic Obstructive Pulmonary Disease; CVD: cardiovascular disease; DM: Diabetes; OR = Odds Ratios; CI: Confidence Intervals; LCL: Lower Confidence Limit; UCL: Upper Confidence Limit. a. 95% CI excluding 1 indicates statistical significance at α: 0.05.

**Figure 4:**
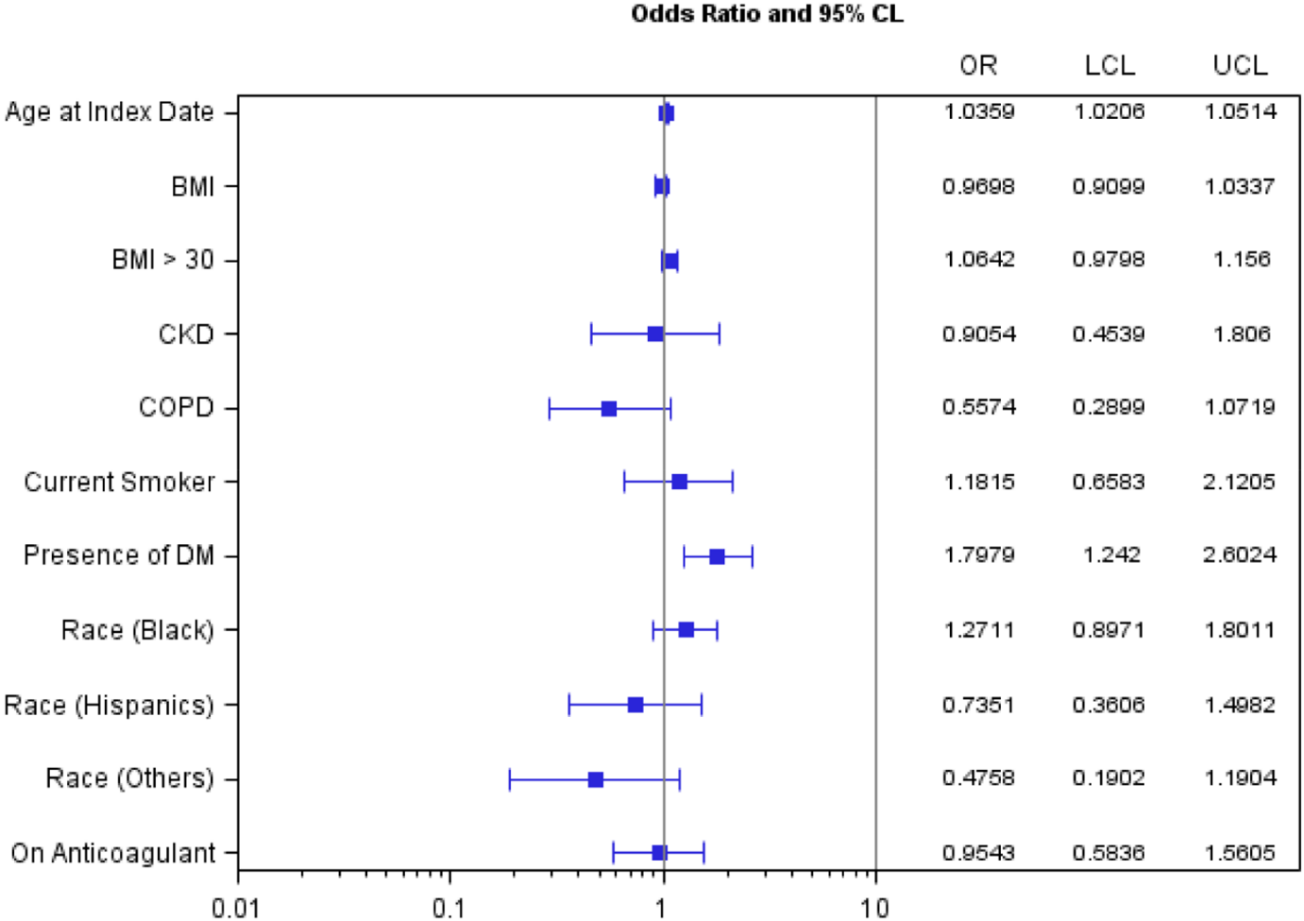
Odds ratios and 95% confidence interval estimates of risk factors for 60-day new diagnosis of heart disease among SARS-CoV-2-positive women veterans. BMI: Body Mass Index; CKD: Chronic Kidney Disease; COPD: Chronic Obstructive Pulmonary Disease; CVD: cardiovascular disease; DM: Diabetes; OR: Odds Ratios; CI: Confidence Intervals; LCL: Lower Confidence Limit; UCL: Upper Confidence Limit. a. 95% CI excluding 1 indicates statistical significance at α = 0.05.

Within the SARS-CoV-2-positive group, older age, obesity, the pre-existing conditions of CVD, CKD, and COPD, and prior antiplatelet or antithrombotic therapy were associated with increased risk of CVD events within 60 days (Fig. 3). Older age and pre-existing diabetes and COPD increased the risk of developing new heart disease after the positive test (Fig. 4).

## Discussion

In this retrospective cohort study in a racially diverse population of women military veterans, we found that women veterans with COVID-19 were at nearly 4 times higher risk of mortality but at a lower risk of experiencing cardiovascular events and developing new heart disease within 60 days compared to those without COVID-19.

The current study confirms older age^4,7,22–24^, obesity^25–27^, and prior CVD^4,14^ and COPD^4,14^ conditions as risk factors for mortality in COVID-19 disease. We observed only weak associations of prior CKD^4,14^ with COVID-19 mortality risk. While SARS-CoV-2 positive women veterans were at lower risk of CVD events at 60 days, positive subjects showed strong associations of older age, obesity, and prior diagnoses of CVD, COPD, and CKD with increased risk of CVD events. Unlike previous studies^14,23,28^, we found that having diabetes prior to a positive test was not a significant risk factor for 60-day mortality^3^, but might increase risk of a new onset of heart disease^29^.

The US veteran population differs from the general population in the prevalence of pre-existing conditions. Moreover, compared to the general population of women, women veterans differ from the general population of women in terms of socio-demographic characteristics. The women veteran population is slightly older (median age 50) than the general population of women (median age 45), and Black women comprise a greater portion of the veteran population (19% vs. 12%). Additionally, Veteran women have a lower rate of being uninsured (4% vs. 9%) and a higher rate of being college educated (35% vs. 28%) and employed (72% vs. 70%)^30^. Overall, minority women veterans may have better access to care than women of minority background in the general population^31^. Nevertheless, the current study, using VA national data, was able to confirm several previously reported risk factors of COVID-19-associated mortality and cardiovascular complications.

Racial and ethnic minority women are understudied in COVID-19 research due to their under-representation in available data sets^10,32^, despite the fact that these groups are disproportionately affected by COVID-19^33^. By focusing on a racially diverse cohort of women veterans, our study can disentangle the effects of race, sex, and access to care on risk factors for COVID-associated mortality and cardiovascular outcomes. Demographic and health data on women veterans are readily available from VA national COVID-19 shared resources built from VA national EHRs. The current study provides a better understanding of this understudied population^10,32^. A further advantage of VA EHR data is that there is no confounding effect of access to care on disease outcomes. Despite the elevated rate of SARS-CoV-2 positivity observed among minority women veterans, there was no accompanying elevated mortality rate. This is in stark contrast to other studies that have suggested that racial and socioeconomic disparities in access to care contribute to the higher mortality rates in minority women diagnosed with COVID^34–38^. Although the cohort of women veterans may not exactly mirror the general population, our study does provide important insight into the effect of COVID on a racially diverse group of women in the absence of confounders such as access to care.

We found that SARS-CoV-2 infection itself does not increase a short-term CVD disease risk in women veterans, which may be explained by the observation that women veterans who tested positive were overall younger and healthier than those who tested negative prior to index date. Although there was an association with early troponin elevation, it is unknown whether this was associated with any cardiac symptoms or EKG changes. Furthermore, as depicted in Fig. 2, there were no increases in new-onset heart disease after 60 days. However, longer-term ramifications of SARS-CoV-2 infection on cardiovascular disease are still unknown. The clinical presentation of COVID-19-associated cardiovascular disease in women may be delayed beyond 60 days. Thus, longer follow-up is warranted.

A history of treatment with either an antiplatelet or antithrombotic medication was consolidated into a single field in the VA COVID-19 shared data repository. Although the current analysis found that there is a significant association between prior antiplatelet or antithrombotic therapy and a cardiovascular event within 60 days of being diagnosed with COVID-19, the lack of granularity in the data collection complicates the interpretation of this result. A history of treatment with an antithrombotic may reflect a patient’s pre-existing risk of deep vein thrombosis or venous thromboembolism, Arterial Fibrillation (A-Fib), or stroke, or a hypercoagulable state prior to COVID-19 infection, which may be associated with worse outcomes^39–41^. Similarly, prior treatment with antiplatelet medications may reflect underlying coronary artery disease or peripheral artery disease. While some clinicians and investigators have proposed anticoagulants as a successful treatment of COVID-19 infected patients with a complication of coagulopathy^42,43^, their effectiveness in improving survival and CVD outcomes is yet to be proven^44,45^. Separate analyses of the role of antiplatelets and antithrombotics in CVD outcomes and mortality after diagnosis with COVID-19, though not feasible with the current data set, would improve our understanding of risk factors driving adverse outcomes in patients with COVID-19. Finally, we did not include post-index pharmacotherapy in a final model to examine factors associated with COVID-19-related mortality or cardiovascular outcomes, since at the time of this writing, there is no single known medication proven to be effective to treat severely ill COVID-19 patients^44^.

Our finding that COVID-19-positive women veterans are much younger and have higher BMI compared to COVID-19 tested negative women veterans may partially reflect that racial and ethnic minority groups had higher prevalence of COVID-19 and were more likely to be obese (Black women veterans)^15^ and younger (Hispanic women veterans)^20^. Thus, it is essential to adjust for these confounding factors, which we have done using Cox and logistic regression models including to test associations with COVID-19 and 60-day mortality and CVD outcomes, respectively.

Our study has some limitations: First, we used CVD event occurrence and new onset of CVD at 60 days as binary outcomes due to data availability; a time-to-event analysis would provide a more detailed picture of the natural history of COVID. Second, the study did not include post-index medications (such as antithrombotics, antiplatelets, angiotensin-converting enzyme inhibitors (ACEI), angiotensin receptor blockers (ARBs), hydroxychloroquine, losartan, and corticosteroids) to examine COVID-associated mortality and cardiovascular complications with respect to pharmacologic interventions, due to limited availability of data on timing and use of concomitant drugs. Third, we cannot rule out human errors in the entry of ICD codes into the VA EHR system; thus, variable construction may be susceptible to coding errors. Additional future studies investigating the long-term consequences of COVID-19 on women’s cardiovascular health are necessary to understand the full societal impact of the COVID-19 pandemic.

## Methods

### Data

**Figure 5:**
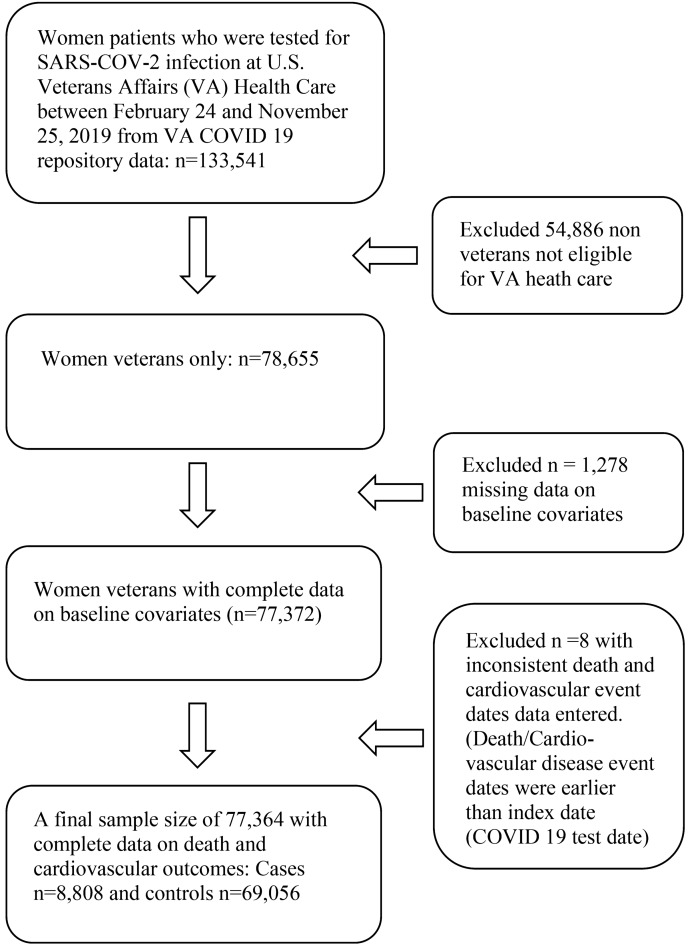
Procedure of inclusion and exclusion of the study data.

We conducted a retrospective cohort study using data on women veterans from a VA COVID-19 shared data resource collected between February and November 2020. The flow chart in Fig. 5 describes a procedure of a final sample selection with inclusion and exclusion criteria. VA COVID-19 shared data included data on patient demographic characteristics, smoking status, and BMI at index date, laboratory testing results, including troponin within 7 days from the date of COVID-19 screen testing (henceforth, the index date), disease conditions and pharmacological therapy up to 2 years prior to and 60-days after the index date, and 60-day adverse events after the index date.

The index date was determined hierarchically. For a patient who ever tested positive (presumed positive) for SARS-CoV-2, the index date was the date of the first positive test. For a patient who tested negative on all occasions, the index date was the date of the first negative test. COVID-19-associated outcomes considered in the current study are all-cause mortality with actual date of death, cardiovascular events, and new onset of cardiovascular disease within 60 days from the index date. Cardiovascular events included non-fatal ischemic and hemorrhagic stroke, myocardial injury defined by troponin level > 0.4 ng/mL within 7 days from index^11^, and non-fatal heart failure. Heart disease included cardiovascular disease, hypertension, coronary artery atherosclerotic heart disease, cerebrovascular disease, cardiomyopathy and other ill-defined heart disease. A new onset of heart disease was defined as any heart disease diagnosed 60 days post index without a prior heart disease condition in the preceding 2 years. Cardiovascular outcome variables were constructed from International Statistical Classification of Disease (ICD) 9 and 10 diagnostic codes from VA EHR (eTable 1 in Supplement). The accuracy of cardiovascular outcome variable construction using ICD codes from VA EHR data for women veterans are reported to be over 92%^20^.

### Baseline characteristics

Patient age, body mass index (BMI), and active smoking status on the index date were collected. Other baseline characteristics included presence of diabetes, chronic kidney disease (CKD), chronic obstructive pulmonary disease (COPD), and a history of treatment with either an antiplatelet drug or antithrombotic drug at any time in the 2 years prior to the index date. Antithrombotic medications included heparin and warfarin (eTable 1 in Supplement).

We analyzed effects of medications administered pre- and post-index hydroxychloroquine, and corticosteroid. All but a history of antiplatelet or antithrombotic drugs were excluded from the final model due to lack of statistical significance. Exclusion of these variables from the final model was guided by Akaike Information Criteria (AIC), multicollinearity tests, and statistical significance.

### Statistical methods

We compared baseline variables and 60-day outcomes between the SARS-CoV-2 positive (+) and negative (–) groups by computing descriptive statistics and *t* and chi-squared tests.

We assessed differences in event-time outcomes between the SARS-CoV-2 positive and negative groups using Kaplan–Meier curves and the Cox proportional hazards model, with adjustment for baseline confounders. and 95% confidence intervals (CI) were presented for Cox model estimates. We censored all event times at 60 days. We assessed differences in binary CVD outcomes between the SARS-CoV-2 positive and negative groups using logistic regression, again with adjustment for baseline confounders. We displayed odds ratios (ORs) from the logistic regressions using forest plots with 95% confidence intervals (CIs).

We conducted all analyses in SAS version 9.4 (SAS Institute, Cary, NC) or R version 3.6.1 (cran.r-project.org).

This study was designated exempt by the VA North Texas Institutional Review Board (IRB), approved by the VA North Texas Research and Development Committee, and supported using data from the VA COVID-19 Shared Data Resource. VA North Texas IRB waived the requirement to obtain informed consents from study subjects.

Because of the sensitive nature of the data collected for this study, requests to access the dataset are limited to qualified VA-affiliated researchers trained in human subject confidentiality. Protocols may be sent to the VA North Texas Health Care System IRB at NTXIRBAdmin@va.gov. SQL, SAS and R code used in the analysis of this study are available from the corresponding author upon reasonable request. All methods were performed in accordance with the relevant guidelines and regulations.

## Supplementary Information

Supplementary Information.
