# Supplementary material for: COVID-19 associated mortality and cardiovascular disease outcomes among US women veterans
Source: Sci Rep. 2021 Apr 19;11:8497. doi: 10.1038/s41598-021-88111-z (PMC8055870; doi:10.1038/s41598-021-88111-z)

Supplement

**COVID-19 associated mortality and cardiovascular disease outcomes among U.S. women veterans**

Shirling Tsai, M.D.^1,2^ ; Hang Nguyen, M.S.^1,3^; Ramin Ebrahimi, M.D.^4,5^; Monica R. Barbosa, M.D.^1,6^; Bala Ramanan, M.B.B.S. ^1,2^; Daniel F. Heitjan, Ph.D.^3,7^; Jeffrey L. Hastings, M.D. ^1,6^; J. Gregory Modrall, M.D. ^1,2^; Haekyung Jeon-Slaughter, Ph.D.^1,6^*

1. Veterans Affairs North Texas Health Care System, Dallas, Texas

2. Department of Surgery, University of Texas Southwestern Medical Center, Dallas Texas

3. Southern Methodist University, Dallas Texas

4. Veterans Affairs Greater Los Angeles Health Care System, Los Angeles, California

5. Department of Medicine, University of California at Los Angeles, Los Angeles, California

6. Department of Internal Medicine, University of Texas Southwestern Medical Center, Dallas, Texas

7. Department of Population and Data Sciences, University of Texas Southwestern Medical Center, Dallas, Texas

*Corresponding author:

Haekyung Jeon-Slaughter, Ph.D.

Statistician, VA North Texas Health Care System

Assistant Professor

Division of Cardiology, Department of Internal Medicine

University of Texas Southwestern Medical Center

4500 South Lancaster Road,

Dallas, Texas 75216

Email: Haekyung.jeon-slaughter@utsouthwestern.edu

Phone (214) 857-3509

**eTable 1. ICD-9 and ICD-10 codes and definitions for variables**

| Variables | Code number | Key definitions |
| --- | --- | --- |
| Hypertension | 401-405, 437, 642; O10, O16 | Hypertensive heart disease, hypertensive chronic kidney disease, hypertensive encephalopathy, hypertension of pregnancy/eclampsia |
| Stroke | 430, 431; I60, I61 | Hemorrhage |
|  | 433; I66 | Occlusion and stenosis of cerebral arteries |
|  | 434; I63 | Cerebral thrombosis; Cerebral infarction |
| COPD | 115 | Histoplasmosis |
|  | 490, 491, 492, 494; J40, J41, J43, J44, J47 | Chronic bronchitis; emphysema |
|  | V81.3 | Screening for chronic bronchitis and emphysema |
| CKD | 361, 364, 458, 792; R88, V45, V56, Z49 | Hemodialysis; renal dialysis status |
|  | 585, 753; N18, V18 | Kidney disease |
| Diabetes mellitus (DM) | 249, 250; E08 – E13, O24, O99 | Diabetes mellitus, Type ½ diabetes mellitus, abnormal glucose complication |
| Cerebrovascular disease | 346 | Migraine aura with cerebral infarction |
|  | 430-432; I60 – I62 | Hemorrhage |
|  | 433; I66, I68 | Occlusion and stenosis of cerebral arteries |
|  | 434 | Cerebral thrombosis; Cerebral infarction |
|  | 435; I67 | Cerebral ischemia |
|  | 436 – 438 | Cerebrovascular disease |
| Cardiomyopathy | 425 | Hypertrophic, alcoholic, nutritional and metabolic cardiomyopathy; other primary/secondary cardiomyopathy |
|  | A36, A38, A39, B26, B33, B58, I40, I42 | Myocarditis; Meningococcal myocarditis, carditis; Viral myocarditis, carditis, cardiomyopathy |
| CAHD | 391 | Acute rheumatic endocarditis, myocarditis |
|  | 411, 414; I24, V17.3, V81 | Acute/chronic ischemic heart diseases; coronary atherosclerosis |
|  | 413, I20 | Angina pectoris |
|  | 425 | Hypertrophic, alcoholic, nutritional and metabolic cardiomyopathy; other primary/secondary cardiomyopathy |
|  | 440; I25 | Atherosclerosis of arteries |
|  | Z95, Z98 | Aortocoronary bypass graft, coronary angioplasty |
| CVD | 410; I20-I24 | Myocardial infarction |
|  | 411, 414 | Acute/chronic ischemic heart diseases |
|  | 428; I50 | Heart failure, acute chronic systolic/diastolic heart failure or combined |
|  | 433, 435; I65 | Occlusion and stenosis of basilar/carotid/vertebral arteries; Transient cerebral ischemia |
|  | 434; I63 | Cerebral embolism/infarction/artery occlusion |
|  | 440; I25, I70 | Atherosclerosis of arteries |
|  | 441 | Dissecting aneurysm; abdominal/aortic/thoracic aneurysm |

Abbreviations. BMI = Body Mass Index; CAHD = coronary artery heart disease; CVD = cardiovascular disease; DM = Diabetes; CKD = Chronic Kidney Disease; COPD = Chronic Obstructive Pulmonary Disease.

**eFigure 1. Proportions of SARS-CoV-2 positive cases out of all women veterans tested by state.**

**
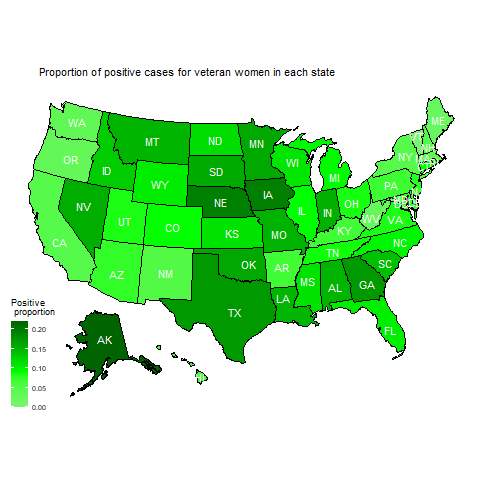
**

**eFigure 2. Numbers of women veterans tested for SARS-CoV-2 and testing positive by month.**

**
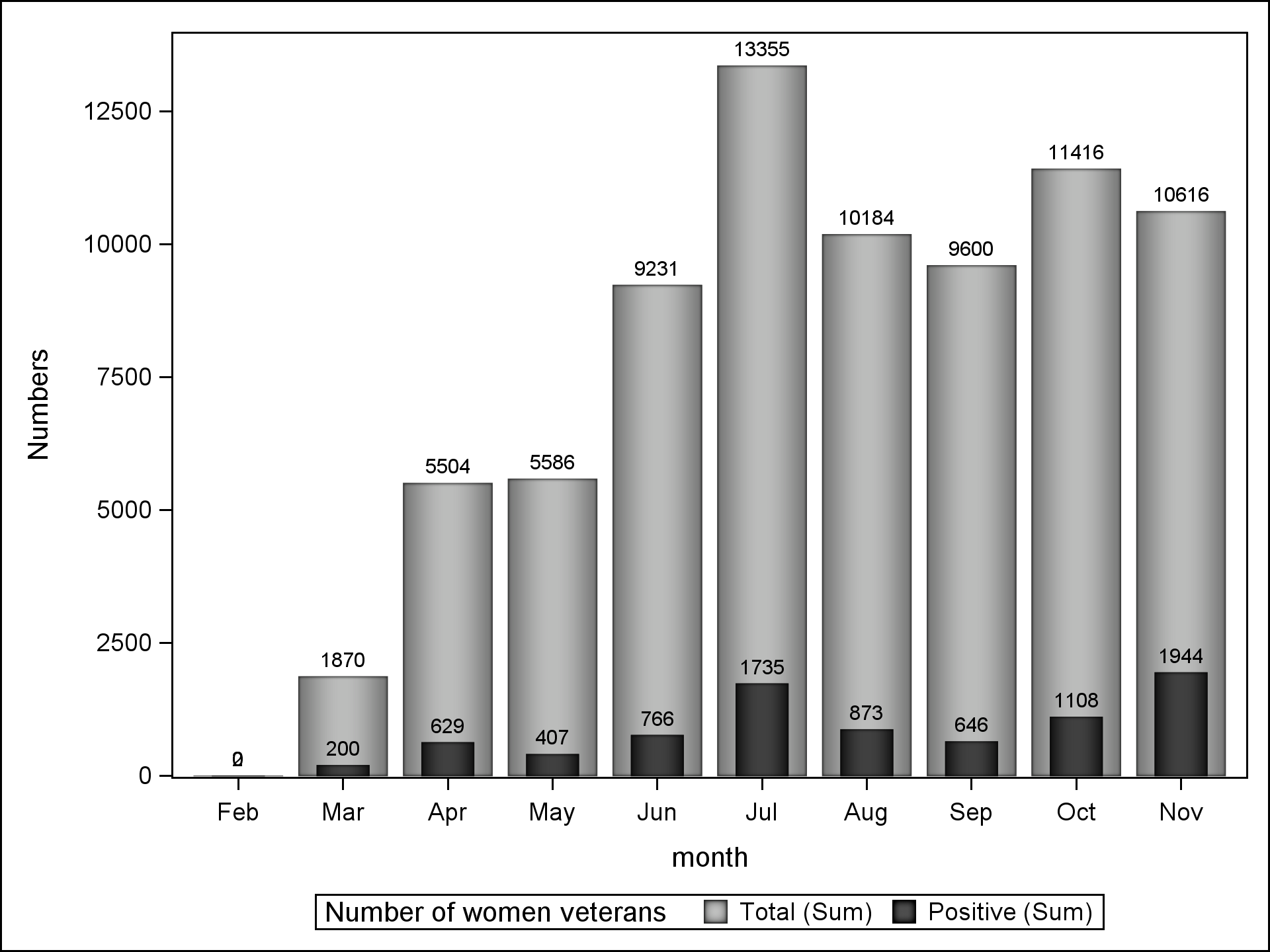
**

**eFigure 3. Number of COVID-19-associated deaths in women veterans by state.**

**
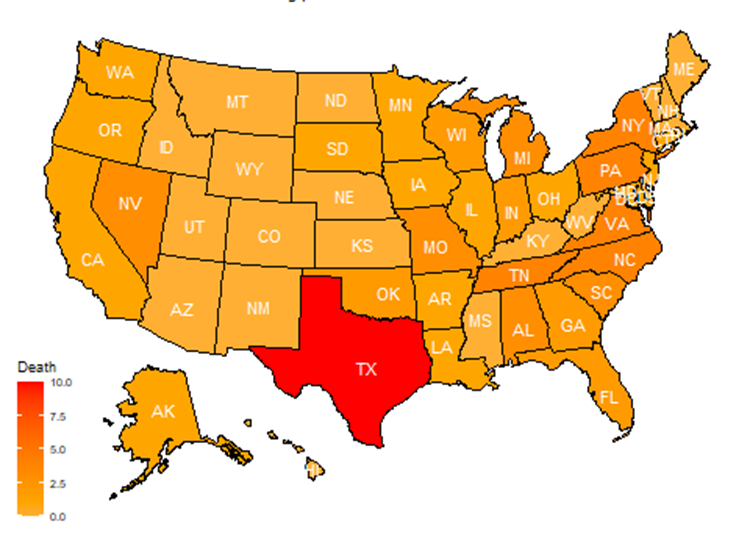
**

**eFigure 4. Body mass Index non-linear spline component results of Generalized Additive Model (GAM) for 60-day mortality risk.**


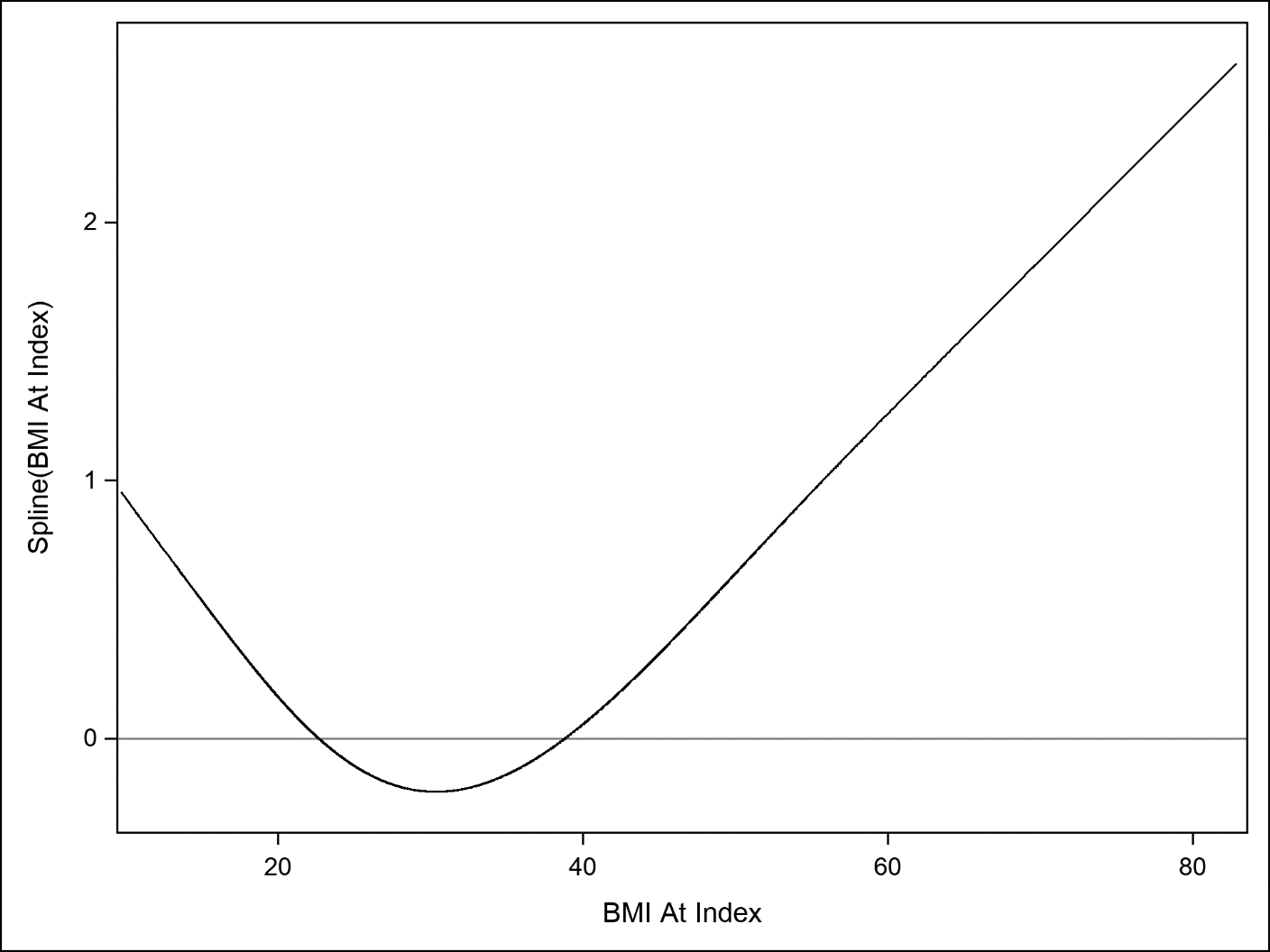

Supplement: Supplementary file 1 — Supplementary Information. [file 41598_2021_88111_MOESM1_ESM.docx]
